# Bis((*E*)-2-{5,5-di­methyl-3-[4-(1*H*-1,2,4-triazol-1-yl-κ*N* ^4^)styr­yl]cyclo­hex-2-enyl­idene}malono­nitrile)­diiodido­mercury(II)

**DOI:** 10.1107/S160053681302518X

**Published:** 2013-09-21

**Authors:** Lian-Ke Wang, Wei-Ju Zhu, Hong-Ping Zhou

**Affiliations:** a Deparment of Chemistry, Anhui University, Hefei 230039, People’s Republic of China and Key Laboratory of Functional Inorganic Materials Chemistry, Hefei 230039, People’s Republic of China

## Abstract

In the title complex, [HgI_2_(C_21_H_19_N_5_)_2_], the Hg^II^ ion is located on a twofold rotation axis and is coordinated by two I atoms and two N atoms from two (*E*)-2-{5,5-di­methyl-3-[4-(1*H*-1,2,4-triazol-1-yl)styr­yl]cyclo­hex-2-enyl­idene}malono­nitrile ligands in a distorted tetra­hedral geometry. In the crystal, the mol­ecules are linked by inter­molecular π–π inter­actions between the triazole and benzene rings [centroid–centroid distance = 3.794 (3) Å] into a band extending in [010]. These bands are further connected by C—H⋯N hydrogen bonds into a two-dimensional network parallel to (100).

## Related literature

For background to metal-organic complexes, see: Haneda *et al.* (2007 ▶); Li *et al.* (2006 ▶); Liu *et al.* (2010 ▶, 2011 ▶); Satapathy *et al.* (2012 ▶); Sun *et al.* (2012 ▶). For the organic ligand of the title compound, see: Zheng *et al.* (2013 ▶). For related structures, see: Jin, Wang *et al.* (2013 ▶); Jin, Zhang *et al.* (2013 ▶); Zhou *et al.* (2009 ▶).
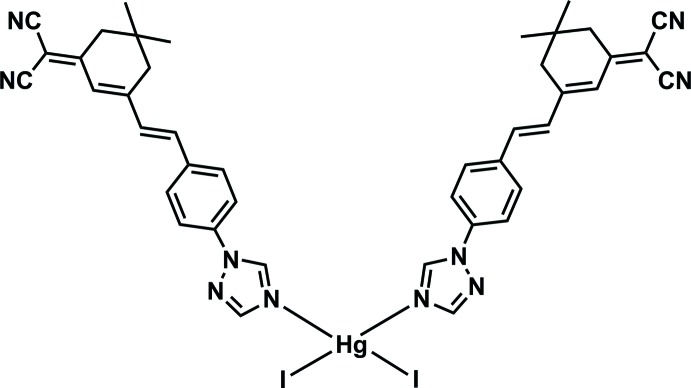


## Experimental

### 

#### Crystal data

[HgI_2_(C_21_H_19_N_5_)_2_]
*M* *_r_* = 1137.21
Monoclinic, 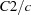

*a* = 38.9622 (16) Å
*b* = 5.5684 (12) Å
*c* = 21.9564 (14) Å
β = 117.738 (2)°
*V* = 4216.2 (10) Å^3^
*Z* = 4
Mo *K*α radiation
μ = 5.16 mm^−1^
*T* = 291 K
0.30 × 0.20 × 0.18 mm

#### Data collection

Bruker APEX CCD diffractometer
Absorption correction: multi-scan (*SADABS*; Bruker, 2001 ▶) *T*_min_ = 0.307, *T*_max_ = 0.457
14961 measured reflections
4078 independent reflections
3384 reflections with *I* > 2σ(*I*)
*R*_int_ = 0.032

#### Refinement

*R*[*F* ^2^ > 2σ(*F* ^2^)] = 0.027
*wR*(*F* ^2^) = 0.071
*S* = 1.01
4078 reflections
251 parameters
H-atom parameters constrained
Δρ_max_ = 0.78 e Å^−3^
Δρ_min_ = −0.52 e Å^−3^

### 

Data collection: *SMART* (Bruker, 2007 ▶); cell refinement: *SAINT* (Bruker, 2007 ▶); data reduction: *SAINT*; program(s) used to solve structure: *SHELXTL* (Sheldrick, 2008 ▶); program(s) used to refine structure: *SHELXTL*; molecular graphics: *XP* in *SHELXTL* and *DIAMOND* (Brandenburg, 1999 ▶); software used to prepare material for publication: *SHELXTL*.

## Supplementary Material

Crystal structure: contains datablock(s) I, Global. DOI: 10.1107/S160053681302518X/hy2634sup1.cif

Structure factors: contains datablock(s) I. DOI: 10.1107/S160053681302518X/hy2634Isup2.hkl

Additional supplementary materials:  crystallographic information; 3D view; checkCIF report

## Figures and Tables

**Table 1:** Hydrogen-bond geometry (Å, °)

*D*—H⋯*A*	*D*—H	H⋯*A*	*D*⋯*A*	*D*—H⋯*A*
C20—H20⋯N2^i^	0.93	2.48	3.354 (7)	157

Symmetry code: (i) 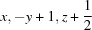.

**Fig. 1.**
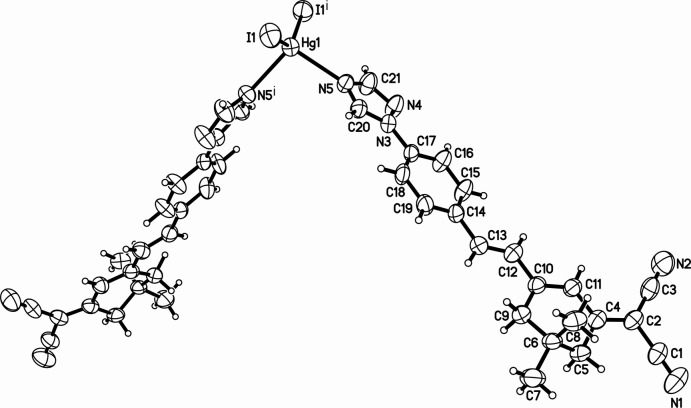
The molecular structure of the title complex. Displacement ellipsoids are drawn at the 50% probability level. [Symmetry code: (i) -x, y, 3/2-z.]

**Fig. 2.**
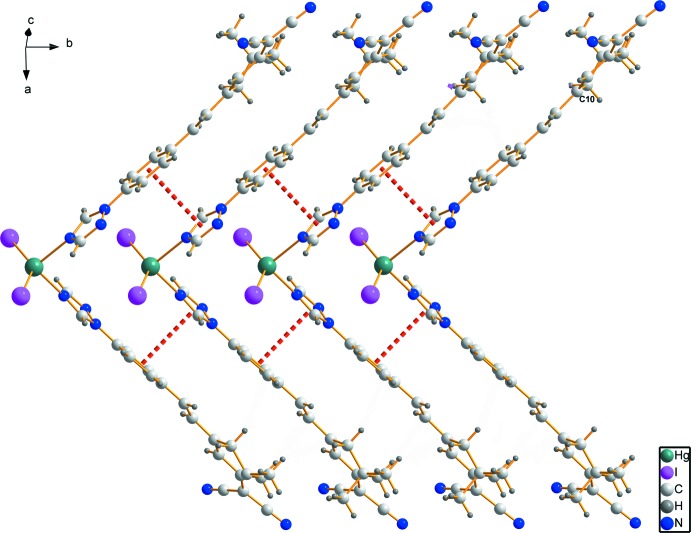
The one-dimensional structure of the title complex, showing π–π interactions (dashed lines).

**Fig. 3.**
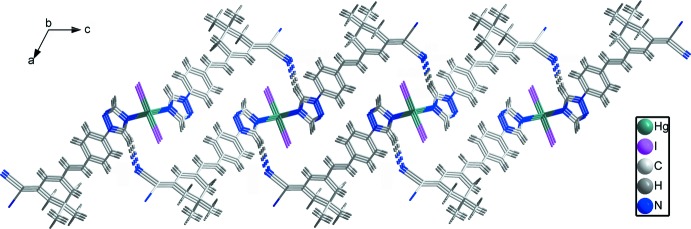
The two-dimensional structure of the title complex, showing C—H···N hydrogen bonds (dashed lines).

## supplementary crystallographic information

### 1. Comment

The design and synthesis of metal-organic hybrid complexes based on strong coordinate bonds and multiple weak non-covalent forces have become one of the most active fields in coordination chemistry and crystal engineering not only for their fascinating structural features but also for their interesting properties as new functional materials with tremendous potential applications in the areas of luminescence, catalysis, separation, adsorption, biological chemistry (Haneda *et al.*, 2007; Li *et al.*, 2006; Liu *et al.*, 2010, 2011; Satapathy *et al.*, 2012; Sun *et al.*, 2012). The organic ligand of the title compound had been investigated for its optical properties (Zheng *et al.*, 2013). A variety of mercury(II) complexes have been reported (Jin, Wang *et al.*, 2013). Besides, triazole and isophorone-malononitrile complexes have been reported (Jin, Zhang *et al.*, 2013; Zhou *et al.*, 2009). In this paper, we report the synthesis and crystal structure of the title complex (Fig. 1). In the crystal, intermolecular π–π interactions between the triazole and benzene rings [centroid–centroid distance = 3.794 (3) Å] link the molecules into a band extending in [010] (Fig. 2). The neighboring bands are further linked into a two-dimensional network parallel to (100) through C—H···N hydrogen bonds (Fig.3).

### 2. Experimental

For the preparation of the title complex, (E)-2-(3-(4-(1*H*-1,2,4-triazol-1-yl)styryl)- 5,5-dimethylcyclohex-2-enylidene)malononitrile (0.341 g, 1 mmol) in 25 ml of dichloromethane was added into a 50 ml colorimeter tube, carefully layered with a clear acetonitrile and benzene solution (25 ml) of HgI_2_ (0.227 g, 0.5 mmol). Crystals were obtained by slow interlayer diffusion (yield: 0.427 g, 75.1%).

### 3. Refinement

All H atoms were placed in geometrically idealized positions and constrained to ride on their parent atoms, with C—H = 0.93 (CH), 0.97 (CH_2_) and 0.96 (CH_3_) Å and with *U*_iso_(H) = 1.2(1.5 for methyl)*U*_eq_(C).

### Crystal data

**Table:**

[HgI_2_(C_21_H_19_N_5_)_2_]	*F*(000) = 2184
*M**_r_* = 1137.21	*D*_x_ = 1.792 Mg m^−^^3^
Monoclinic, *C*2/*c*	Mo *K*α radiation, λ = 0.71073 Å
*a* = 38.9622 (16) Å	Cell parameters from 3126 reflections
*b* = 5.5684 (12) Å	θ = 2.1–23.6°
*c* = 21.9564 (14) Å	µ = 5.16 mm^−^^1^
β = 117.738 (2)°	*T* = 291 K
*V* = 4216.2 (10) Å^3^	Needle, yellow
*Z* = 4	0.30 × 0.20 × 0.18 mm

### Data collection

**Table:**

Bruker APEX CCD diffractometer	4078 independent reflections
Radiation source: fine-focus sealed tube	3384 reflections with *I* > 2σ(*I*)
Graphite monochromator	*R*_int_ = 0.032
φ and ω scans	θ_max_ = 26.0°, θ_min_ = 1.9°
Absorption correction: multi-scan (*SADABS*; Bruker, 2001)	*h* = −47→47
*T*_min_ = 0.307, *T*_max_ = 0.457	*k* = −6→6
14961 measured reflections	*l* = −26→26

### Refinement

**Table:**

Refinement on *F*^2^	Primary atom site location: structure-invariant direct methods
Least-squares matrix: full	Secondary atom site location: difference Fourier map
*R*[*F*^2^ > 2σ(*F*^2^)] = 0.027	Hydrogen site location: inferred from neighbouring sites
*wR*(*F*^2^) = 0.071	H-atom parameters constrained
*S* = 1.01	*w* = 1/[σ^2^(*F*_o_^2^) + (0.04*P*)^2^ + 0.22*P*] where *P* = (*F*_o_^2^ + 2*F*_c_^2^)/3
4078 reflections	(Δ/σ)_max_ = 0.001
251 parameters	Δρ_max_ = 0.78 e Å^−^^3^
0 restraints	Δρ_min_ = −0.52 e Å^−^^3^

### Special details

**Table:**

Geometry. All e.s.d.'s (except the e.s.d. in the dihedral angle between two l.s. planes) are estimated using the full covariance matrix. The cell e.s.d.'s are taken into account individually in the estimation of e.s.d.'s in distances, angles and torsion angles; correlations between e.s.d.'s in cell parameters are only used when they are defined by crystal symmetry. An approximate (isotropic) treatment of cell e.s.d.'s is used for estimating e.s.d.'s involving l.s. planes.

### Fractional atomic coordinates and isotropic or equivalent isotropic displacement parameters (Å^2^)

**Table:**

	*x*	*y*	*z*	*U*_iso_*/*U*_eq_	
Hg1	0.0000	1.49817 (4)	0.7500	0.04679 (9)	
I1	0.06739 (2)	1.65983 (6)	0.84809 (2)	0.06479 (11)	
C19	0.11064 (13)	0.4748 (8)	0.6784 (2)	0.0541 (11)	
H19	0.1329	0.4209	0.7164	0.065*	
C15	0.06588 (13)	0.4500 (8)	0.5599 (2)	0.0560 (11)	
H15	0.0575	0.3788	0.5170	0.067*	
C16	0.04479 (12)	0.6381 (8)	0.5667 (2)	0.0540 (10)	
H16	0.0227	0.6937	0.5288	0.065*	
N5	0.01215 (9)	1.2154 (6)	0.67843 (16)	0.0452 (7)	
N3	0.03392 (9)	0.9312 (6)	0.63805 (16)	0.0409 (7)	
C13	0.12305 (12)	0.1699 (7)	0.6110 (2)	0.0485 (9)	
H13	0.1406	0.1013	0.6525	0.058*	
C21	−0.01308 (12)	1.1596 (8)	0.6130 (2)	0.0590 (11)	
H21	−0.0370	1.2354	0.5892	0.071*	
C20	0.04150 (12)	1.0708 (7)	0.6927 (2)	0.0447 (9)	
H20	0.0641	1.0659	0.7344	0.054*	
C17	0.05698 (10)	0.7431 (6)	0.63072 (19)	0.0401 (8)	
C14	0.09914 (11)	0.3646 (7)	0.6153 (2)	0.0439 (9)	
N4	−0.00144 (11)	0.9900 (6)	0.58534 (19)	0.0614 (10)	
C18	0.08964 (12)	0.6650 (8)	0.6862 (2)	0.0528 (10)	
H18	0.0979	0.7374	0.7290	0.063*	
C10	0.14855 (11)	−0.1065 (7)	0.5526 (2)	0.0430 (9)	
C9	0.17746 (11)	−0.2203 (7)	0.61823 (19)	0.0481 (9)	
H9A	0.1640	−0.3332	0.6333	0.058*	
H9B	0.1885	−0.0966	0.6532	0.058*	
C11	0.14662 (11)	−0.1692 (7)	0.4915 (2)	0.0468 (9)	
H11	0.1295	−0.0878	0.4520	0.056*	
C12	0.12256 (12)	0.0798 (7)	0.5548 (2)	0.0490 (10)	
H12	0.1040	0.1402	0.5129	0.059*	
C6	0.21068 (11)	−0.3527 (6)	0.61357 (19)	0.0451 (9)	
C5	0.19376 (13)	−0.5051 (6)	0.5484 (2)	0.0504 (10)	
H5A	0.2147	−0.5826	0.5437	0.061*	
H5B	0.1775	−0.6298	0.5524	0.061*	
C4	0.17029 (11)	−0.3582 (7)	0.4857 (2)	0.0448 (9)	
C1	0.19109 (13)	−0.6083 (9)	0.4172 (2)	0.0556 (10)	
C2	0.17018 (12)	−0.4045 (8)	0.4249 (2)	0.0493 (10)	
C3	0.14937 (13)	−0.2621 (9)	0.3649 (2)	0.0566 (11)	
N2	0.13295 (13)	−0.1425 (8)	0.3170 (2)	0.0772 (12)	
N1	0.20731 (13)	−0.7689 (8)	0.4121 (2)	0.0824 (13)	
C8	0.24041 (12)	−0.1776 (7)	0.6121 (2)	0.0580 (11)	
H8A	0.2609	−0.2658	0.6095	0.087*	
H8B	0.2510	−0.0819	0.6531	0.087*	
H8C	0.2280	−0.0748	0.5726	0.087*	
C7	0.23118 (16)	−0.5185 (7)	0.6758 (3)	0.0698 (14)	
H7A	0.2131	−0.6351	0.6761	0.105*	
H7B	0.2411	−0.4252	0.7173	0.105*	
H7C	0.2522	−0.5992	0.6732	0.105*	

### Atomic displacement parameters (Å^2^)

**Table:**

	*U*^11^	*U*^22^	*U*^33^	*U*^12^	*U*^13^	*U*^23^
Hg1	0.05187 (14)	0.04752 (14)	0.04360 (14)	0.000	0.02444 (11)	0.000
I1	0.0631 (2)	0.0737 (2)	0.05467 (19)	−0.01842 (15)	0.02500 (15)	−0.00968 (15)
C19	0.056 (3)	0.069 (3)	0.043 (2)	0.019 (2)	0.027 (2)	0.012 (2)
C15	0.053 (3)	0.064 (3)	0.050 (3)	0.000 (2)	0.023 (2)	−0.023 (2)
C16	0.044 (2)	0.066 (3)	0.046 (2)	0.008 (2)	0.0154 (18)	−0.016 (2)
N5	0.0489 (19)	0.0457 (18)	0.0442 (18)	0.0023 (15)	0.0242 (16)	−0.0050 (15)
N3	0.0427 (18)	0.0461 (17)	0.0376 (17)	0.0000 (14)	0.0219 (15)	−0.0028 (14)
C13	0.056 (2)	0.045 (2)	0.053 (2)	0.0027 (18)	0.032 (2)	0.0038 (19)
C21	0.048 (2)	0.077 (3)	0.048 (2)	0.014 (2)	0.019 (2)	−0.007 (2)
C20	0.049 (2)	0.047 (2)	0.039 (2)	0.0000 (18)	0.0210 (18)	−0.0038 (17)
C17	0.046 (2)	0.0409 (19)	0.043 (2)	−0.0022 (17)	0.0285 (18)	−0.0026 (17)
C14	0.049 (2)	0.045 (2)	0.049 (2)	−0.0011 (18)	0.0314 (19)	−0.0004 (18)
N4	0.050 (2)	0.082 (3)	0.044 (2)	0.0147 (18)	0.0156 (17)	−0.0155 (18)
C18	0.064 (3)	0.065 (3)	0.035 (2)	0.016 (2)	0.028 (2)	−0.001 (2)
C10	0.047 (2)	0.0361 (19)	0.051 (2)	−0.0018 (17)	0.0268 (19)	−0.0027 (18)
C9	0.057 (2)	0.041 (2)	0.051 (2)	−0.0002 (18)	0.029 (2)	−0.0021 (19)
C11	0.050 (2)	0.045 (2)	0.045 (2)	0.0068 (18)	0.0219 (19)	−0.0007 (18)
C12	0.048 (2)	0.048 (2)	0.055 (3)	0.0043 (19)	0.027 (2)	−0.004 (2)
C6	0.055 (2)	0.0315 (19)	0.045 (2)	0.0024 (17)	0.0202 (18)	0.0003 (17)
C5	0.061 (3)	0.032 (2)	0.061 (3)	0.0023 (18)	0.031 (2)	−0.0009 (19)
C4	0.042 (2)	0.042 (2)	0.053 (2)	−0.0049 (17)	0.0240 (18)	−0.0077 (18)
C1	0.056 (3)	0.056 (3)	0.060 (3)	−0.002 (2)	0.031 (2)	−0.014 (2)
C2	0.047 (2)	0.045 (2)	0.054 (3)	−0.0012 (19)	0.023 (2)	−0.012 (2)
C3	0.053 (3)	0.070 (3)	0.045 (3)	0.000 (2)	0.021 (2)	−0.018 (2)
N2	0.081 (3)	0.087 (3)	0.055 (3)	0.007 (2)	0.024 (2)	−0.007 (2)
N1	0.082 (3)	0.075 (3)	0.103 (3)	0.003 (2)	0.053 (3)	−0.028 (3)
C8	0.051 (2)	0.047 (2)	0.071 (3)	−0.0008 (19)	0.024 (2)	−0.002 (2)
C7	0.091 (4)	0.048 (3)	0.062 (3)	0.014 (2)	0.028 (3)	0.009 (2)

### Geometric parameters (Å, º)

**Table:**

Hg1—N5	2.422 (3)	C10—C9	1.495 (5)
Hg1—I1	2.6606 (8)	C9—C6	1.533 (5)
C19—C14	1.385 (6)	C9—H9A	0.9700
C19—C18	1.397 (5)	C9—H9B	0.9700
C19—H19	0.9300	C11—C4	1.444 (5)
C15—C16	1.381 (5)	C11—H11	0.9300
C15—C14	1.384 (6)	C12—H12	0.9300
C15—H15	0.9300	C6—C5	1.524 (5)
C16—C17	1.386 (5)	C6—C8	1.526 (5)
C16—H16	0.9300	C6—C7	1.531 (6)
N5—C20	1.312 (5)	C5—C4	1.494 (6)
N5—C21	1.346 (5)	C5—H5A	0.9700
N3—C20	1.342 (5)	C5—H5B	0.9700
N3—N4	1.364 (5)	C4—C2	1.357 (5)
N3—C17	1.437 (5)	C1—N1	1.131 (5)
C13—C12	1.324 (5)	C1—C2	1.452 (6)
C13—C14	1.461 (5)	C2—C3	1.424 (6)
C13—H13	0.9300	C3—N2	1.153 (5)
C21—N4	1.313 (5)	C8—H8A	0.9600
C21—H21	0.9300	C8—H8B	0.9600
C20—H20	0.9300	C8—H8C	0.9600
C17—C18	1.360 (5)	C7—H7A	0.9600
C18—H18	0.9300	C7—H7B	0.9600
C10—C11	1.354 (5)	C7—H7C	0.9600
C10—C12	1.466 (5)		
			
N5^i^—Hg1—N5	98.89 (15)	C10—C9—H9A	108.6
N5^i^—Hg1—I1	96.43 (8)	C6—C9—H9A	108.6
N5—Hg1—I1	109.14 (7)	C10—C9—H9B	108.6
N5^i^—Hg1—I1^i^	109.15 (7)	C6—C9—H9B	108.6
N5—Hg1—I1^i^	96.43 (8)	H9A—C9—H9B	107.5
I1—Hg1—I1^i^	140.450 (19)	C10—C11—C4	122.2 (4)
C14—C19—C18	121.6 (4)	C10—C11—H11	118.9
C14—C19—H19	119.2	C4—C11—H11	118.9
C18—C19—H19	119.2	C13—C12—C10	126.0 (4)
C16—C15—C14	121.7 (4)	C13—C12—H12	117.0
C16—C15—H15	119.1	C10—C12—H12	117.0
C14—C15—H15	119.1	C5—C6—C8	109.7 (3)
C15—C16—C17	119.3 (4)	C5—C6—C7	108.7 (3)
C15—C16—H16	120.4	C8—C6—C7	108.6 (4)
C17—C16—H16	120.4	C5—C6—C9	108.7 (3)
C20—N5—C21	103.6 (3)	C8—C6—C9	111.5 (3)
C20—N5—Hg1	131.0 (3)	C7—C6—C9	109.6 (4)
C21—N5—Hg1	125.2 (3)	C4—C5—C6	111.9 (3)
C20—N3—N4	109.6 (3)	C4—C5—H5A	109.2
C20—N3—C17	129.3 (3)	C6—C5—H5A	109.2
N4—N3—C17	121.1 (3)	C4—C5—H5B	109.2
C12—C13—C14	127.5 (4)	C6—C5—H5B	109.2
C12—C13—H13	116.3	H5A—C5—H5B	107.9
C14—C13—H13	116.3	C2—C4—C11	121.1 (4)
N4—C21—N5	115.0 (4)	C2—C4—C5	121.6 (4)
N4—C21—H21	122.5	C11—C4—C5	117.3 (3)
N5—C21—H21	122.5	N1—C1—C2	178.8 (5)
N5—C20—N3	109.7 (4)	C4—C2—C3	122.9 (4)
N5—C20—H20	125.1	C4—C2—C1	121.2 (4)
N3—C20—H20	125.1	C3—C2—C1	115.9 (4)
C18—C17—C16	120.7 (3)	N2—C3—C2	178.6 (5)
C18—C17—N3	120.3 (3)	C6—C8—H8A	109.5
C16—C17—N3	119.0 (3)	C6—C8—H8B	109.5
C15—C14—C19	117.5 (3)	H8A—C8—H8B	109.5
C15—C14—C13	124.2 (4)	C6—C8—H8C	109.5
C19—C14—C13	118.3 (4)	H8A—C8—H8C	109.5
C21—N4—N3	102.1 (3)	H8B—C8—H8C	109.5
C17—C18—C19	119.2 (4)	C6—C7—H7A	109.5
C17—C18—H18	120.4	C6—C7—H7B	109.5
C19—C18—H18	120.4	H7A—C7—H7B	109.5
C11—C10—C12	119.6 (4)	C6—C7—H7C	109.5
C11—C10—C9	121.0 (3)	H7A—C7—H7C	109.5
C12—C10—C9	119.4 (3)	H7B—C7—H7C	109.5
C10—C9—C6	114.9 (3)		
			
C14—C15—C16—C17	−0.6 (7)	N3—C17—C18—C19	177.2 (3)
C20—N5—C21—N4	−0.1 (5)	C14—C19—C18—C17	0.4 (6)
Hg1—N5—C21—N4	−175.7 (3)	C11—C10—C9—C6	16.2 (5)
C21—N5—C20—N3	−0.8 (4)	C12—C10—C9—C6	−162.3 (3)
Hg1—N5—C20—N3	174.5 (2)	C12—C10—C11—C4	−177.3 (4)
N4—N3—C20—N5	1.4 (5)	C9—C10—C11—C4	4.1 (6)
C17—N3—C20—N5	−178.0 (3)	C14—C13—C12—C10	176.2 (4)
C15—C16—C17—C18	1.0 (6)	C11—C10—C12—C13	−175.1 (4)
C15—C16—C17—N3	−177.2 (4)	C9—C10—C12—C13	3.4 (6)
C20—N3—C17—C18	8.1 (6)	C10—C9—C6—C5	−45.2 (4)
N4—N3—C17—C18	−171.2 (4)	C10—C9—C6—C8	75.8 (4)
C20—N3—C17—C16	−173.8 (4)	C10—C9—C6—C7	−163.9 (4)
N4—N3—C17—C16	6.9 (5)	C8—C6—C5—C4	−66.4 (4)
C16—C15—C14—C19	0.1 (6)	C7—C6—C5—C4	175.1 (4)
C16—C15—C14—C13	−178.7 (4)	C9—C6—C5—C4	55.8 (4)
C18—C19—C14—C15	0.0 (6)	C10—C11—C4—C2	−174.5 (4)
C18—C19—C14—C13	178.9 (4)	C10—C11—C4—C5	7.5 (6)
C12—C13—C14—C15	15.6 (7)	C6—C5—C4—C2	143.5 (4)
C12—C13—C14—C19	−163.3 (4)	C6—C5—C4—C11	−38.5 (5)
N5—C21—N4—N3	0.9 (5)	C11—C4—C2—C3	4.7 (6)
C20—N3—N4—C21	−1.3 (4)	C5—C4—C2—C3	−177.4 (4)
C17—N3—N4—C21	178.1 (3)	C11—C4—C2—C1	−174.2 (4)
C16—C17—C18—C19	−0.9 (6)	C5—C4—C2—C1	3.7 (6)

Symmetry code: (i) −*x*, *y*, −*z*+3/2.

### Hydrogen-bond geometry (Å, º)

**Table:**

*D*—H···*A*	*D*—H	H···*A*	*D*···*A*	*D*—H···*A*
C20—H20···N2^ii^	0.93	2.48	3.354 (7)	157

Symmetry code: (ii) *x*, −*y*+1, *z*+1/2.
